# A case of bi-ventricular extensive calcification caused by multiple factors

**DOI:** 10.1186/s12887-020-1973-x

**Published:** 2020-02-22

**Authors:** Xiaoyan Tu, Zhihui Hu, Kevin Yang, Zhuoqing Hu, Yi Jiang

**Affiliations:** 1 Dept of Pediatrics, Renmin Hospital of Wuhan University , Wuhan, 430060 Hubei Province People’s Republic of China; 2 Dept of Endocrinology, The second affiliated hospital of Guangzhou Medical University, Guangzhou, 510000 Guangdong Province People’s Republic of China; 3 Dept of The first clinical medical college, Guangdong medical University, Zhanjiang, 524000 Guangdong Province People’s Republic of China; 4 Dept of Cardiology, Sun Yat-sen University, Guangzhou, 510000 Guangdong Province People’s Republic of China

**Keywords:** Myocardial calcification, Chest computerized tomography, Multiple organ dysfunction syndrome, Disseminated intravascular coagulation

## Abstract

**Background:**

Extensive myocardial calcification has a low incidence rate, but when the patients do have extensive myocardial cases, the prognosis is usually poor. Several sepsis-related extensive myocardial calcification cases have been reported, but there are cases of biventricular calcifications that are caused by multiple cases besides bacteremia and the treatment for it has a low percentage of success.

**Case presentation:**

A 9 year old girl had an extensive biventricular calcification which is caused by multiple factors including multiple organ failure (heart, lung, liver, and kidney), aseptic cardiomyopathy, systemic inflammatory response syndrome, pulmonary hemorrhage, viral encephalitis. In this case study, the massive myocardial calcification present in the patient was classified as dystrophic. After the patient was transferred to the Intensive care unit, a series of rescue treatments such as anti-inflammatory factor storm were implemented to protect the organs. In the end, the patient was rescued from the rescue treatment procedure. After 18 months of follow-up, it was observed that the patient’s heart function returned to normal and it was observed that there was no change in myocardial calcification in the patient.

**Conclusion:**

In this case study, it showcased a case of the diffused biventricular calcification that caused by multiple factors. Furthermore, the precise role of calcification on cardiac function was largely unknown and there has to be further follow-up observation on the patient.

## Background

Extensive myocardial calcification is a rare clinical phenomenon and the prognosis of the extensive myocardial calcification is usually poor. Pathologic calcification in any tissue represents abnormal accumulation of calcium salts. Dystrophic and metastatic are recognized as two basic calcification forms. Dystrophic calcification represents the sequelae of local tissue damage and cellular necrosis. It is not associated with abnormalities in serum calcium levels or calcium homeostasis; however, hypercalcemia will accentuate the process Metastatic calcification represents the sequelae of a systemic process-hypercalcemia and/or abnormalities of calcium homeostasis-and can occur in normal or diseased tissue [1].

The 2 most common outcomes of extensive myocardial calcification are respectively death and myocardial dysfunction. According to previous reports of myocardial calcification, when a large amount of catecholamine was given to a patient under hypotension caused by septic shock, the large amount of catecholamine induced myocardial damage in the patient. The myocardial damage in the patient would lead to cardiomyopathy and that would eventually lead to calcification [2]. Previous reports had also reported that chest imaging had detected the presence of myocardial calcification in critically ill patients under toxic shock syndrome [3].

## Case presentation

**Fig. 1:**
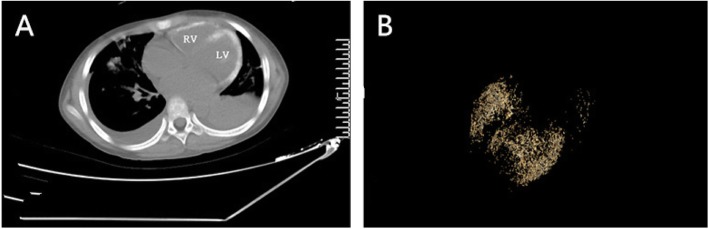
**a** Routine computed tomography(CT) scan of the heart showing extensive calcification of both ventricular walls and chordae on the 14th day; **b** Revolution CT scanning of the myocardial calcification

A 9 years old girl was admitted to a general pediatrics department and she showed symptoms of cough, vomit, abdominal pain, and twitching. The patient rapidly developed severe hypoxemia (SpO2: 45–70%), dyspnea, and heart failure on the next day. She was then transferred to the ICU (Intensive care unit), initial echocardiography at ICU showed enlargement of the left atrium and left ventricle, the magnitudes of ventricular wall motion was lower with an estimated 36% ejection fraction. A series of rescue treatment were implemented such as Continuous renal replacement therapy, large doses of dexamethasone impact therapy, and antibiotics. Most surprisingly is that the initial Chest CT (Computerized tomography) scan revealed extensive calcification of both ventricular walls and chordate on the 14th day (Fig. 1a.), which was confirmed by Cardiac Revolution CT scan subsequently (Fig. 1b). As her condition was improving, the follow-up echocardiography on the 16th day demonstrated that the ejection fraction of the left ventricular had recovered to 50%. A series of diagnosis was established including multiple organ failure (heart, lung, liver, and kidney), septic cardiomyopathy, systemic inflammatory response syndrome, pulmonary hemorrhage, viral encephalitis, myocardial calcification, bacterial pneumonia, electrolyte disorder (hyperkalemia, hyponatremia, hypocalcemia), metabolic acidosis, and disseminated intravascular coagulation.

Compared to treatments in other case of myocardial calcification, there were negligible difference in standard treatments that were carried out in this case study. However, it is summarized that the favorable outcome in the patient was mainly attributed to the series of rescue treatments that were carried out when the MODS (Multiple organ dysfunction syndrome) appeared, the advantages and disadvantages for all the treatments that were implemented to protect the organs were took in account. During the intensive care unit admission, every vital signs were recorded every 1 h and the dose of antibiotics were calculated according to the weight of the child. The dose of antibiotics were used with half a dose of norepinephrine which was only used only one time. The dose of norepinephrine was given by the lowest amount which is 12 mg because when the patient is in hypotension shock, the norepinephrine can help avoid catecholamine-ranted ischemia. When considering that the current occurrence of MODS may be related to the storm of inflammatory factors, methylprednisolone pulse therapy (500 mg QD) was used. The dose was reduced and maintained after 3 days, and stopped when the vital signs were normal and stable.

Furthermore, there was a follow-up on the patient for 18 months and the Magnetic Resonance Imaging images showed no changes in the myocardial calcification, showing that the patients didn’t have further extensive myocardial calcification. The cardiac function and the wall motion of the patient had also completely recovered in the 18 months follow-up. The follow-up echocardiography showed that the patient’s condition and cardiac function were completely normal.

## Discussion and conclusions

Extensive myocardial calcification is a rare clinical phenomenon and the 2 most common outcomes of extensive myocardial calcification are respectively death and myocardial dysfunction. A rare case of calcification of both ventricles that were caused by multiple factors and the outcome of patient from a long term follow-up can be seen in this case study.

In this case study, the massive myocardial calcification present in the patient was classified as dystrophic. Unlike other cases of myocardial calcifications that were mostly only affiliated with sepsis, the myocardial calcification in the patient was affiliated with many other pathologies besides including multiple organ dysfunction syndrome (heart, kidney, lung, and liver), systemic inflammatory response syndrome, disseminated intravascular coagulation, septic cardiomyopathy, and metabolic acidosis. A pathogenesis of sepsis-related myocardial calcification has been hypothesized that a complex inflammatory network response might be the primary mechanism causing the myocardial calcification [4]. In animal models that were under sepsis, calcium overload of cardiomyocytes were detected which suggested in myocardial depression [5]. In this case study, the continuous elevation of myocardial enzymes, inflammatory indicators, reduced ejection fraction and reduced contractility of the myocardium supported in myocardial depression in the patient. There are no reports on DIC (Disseminated intravascular coagulation) patients with myocardial calcification and the question of whether DIC may contribute to myocardial calcification is largely unknown. Although the patient in this case study had acquired pneumonia during the intensive care unit admission and there had been reports of pneumonia-related sepsis in major myocardial calcification cases, it can be hypothesized that the inflammatory factor storm secondary to viral encephalitis was the primary cause that leads to subsequent diseases including septic cardiomyopathy.

Based on previous reports, the diffused myocardial calcification is a life-threatening pathology and has a high mortality rate. In myocardial calcification that is associated with multiple organ dysfunction syndrome, both the 34-year-old male patient reported by Lapatto Reiniluoto and the 72-year-old male patient reported by Aniello Maiese had died [2, 6]. There were no articles reported that the calcified myocardium and myocardial depression were completely reversible in the surviving patients. Furthermore, there are little information about the impact of extensive myocardial on the cardiac function in long term follow-up (over 18 months). As Kapandji N reported, the calcification on patient was stable and the patient had persistent diastolic dysfunction 18 months after discharge [7]. In this case study, the patient not only survived after the treatment, but the cardiac function and wall motion had completely recovered after 18 months. The precise role of the calcification on cardiac function still, however, needs further follow-up observation.

Extensive myocardial calcification diagnosis should be in mind when a patient presents acute myocardial injury accompanied by a series of complication. The chest CT scan is the most valuable tool for diagnosing myocardial calcification and should be used in routine examination. Although the diffused calcifications caused by combinations of factors that are secondary to viral encephalitis is rare in incidence rate and high in mortality rate, there was a successful treatment in this case study. The myocardial calcification had no significant change from initial discovery after the 18 months follow-up. However, the precise role of calcification on cardiac function need further follow-up observation. In addition, the therapeutic intervention method we use does not exclude the existence of individualization, and its potential mechanism of action and whether its widespread clinical application is effective still need to be observed.

## Data Availability

All data generated or analyzed during this study are included in this published article.

## Abbreviations

CT: Computerized tomography
DIC: Disseminated intravascular coagulation
ICU: Intensive care unit
MODS: Multiple organ dysfunction syndrome
